# Genetic diversity, population structure, and gene flow analysis of lowland bamboo [*Oxytenanthera abyssinica* (A. Rich.) Munro] in Ethiopia

**DOI:** 10.1002/ece3.6762

**Published:** 2020-09-20

**Authors:** Oumer Abdie Oumer, Kifle Dagne, Tileye Feyissa, Kassahun Tesfaye, Jayaraman Durai, Muhammad Zeeshan Hyder

**Affiliations:** ^1^ Department of Microbial, Cellular and Molecular Biology Addis Ababa University (AAU) Addis Ababa Ethiopia; ^2^ Department of Biology Assosa University (ASU) Assosa Ethiopia; ^3^ Institute of Biotechnology (IoB) Addis Ababa University (AAU) Addis Ababa Ethiopia; ^4^ Ethiopian Biotechnology Institute (EBTi) Ministry of Science and Technology (MoST) Addis Ababa Ethiopia; ^5^ International Network for Bamboo and Rattan (INBAR) East Africa Regional Office (EARO) Addis Ababa Ethiopia; ^6^ Department of Biosciences COMSATS University Islamabad (CUI) Islamabad Pakistan

**Keywords:** bamboo, genetic differentiation, ISSR primers, *Oxytenanthera abyssinica*, population structure

## Abstract

Bamboo, a member of subfamily Bambusoideae in the grass family (Poaceae), is one of the most important nontimber forest resources and a potential alternative to wood and wood products. Ethiopian lowland bamboo (*Oxytenanthera abyssinica*) is an economically and ecologically important species which accounts about 85% of total bamboo coverage in the country. This species is experiencing population decline due to a number of anthropogenic factors. As a foundation step, genetic diversity, population structure, and gene flow analysis of various *O. abyssinica* populations found in Ethiopia are studied using inter‐simple sequence repeat markers. One hundred and thirty isolates of bamboo belonging to 13 geographically diverse populations were collected for DNA extraction and analysis. Heterozygosity, level of polymorphism, marker efficiency, Nei's gene diversity (*H*), and Shannon's information index (*I*) analysis, analysis of molecular variance (AMOVA), analysis for cluster, principal coordinates (PCoA), and admixture analyses were performed based on the markers banding pattern. The results indicated high genetic variation (84.48%) at species level. The *H*, *I*, observed and effective number of alleles at the species level were 0.2702, 0.4061, 1.8448, and 1.4744, respectively, suggesting a relatively high level of genetic diversity. However, genetic differentiation at the population level was relatively low. Using grouped populations, AMOVA revealed that most (61.05%) of the diversity was distributed within the populations with *F*_ST_ = 0.38949, *F*_SC_ = 0.10486, and *F*_CT_ = 0.31797. Cluster analysis grouped the populations into markedly distinct clusters, suggesting confined propagation in distinct geographic regions. STRUCTURE analyses showed *K* = 2 for all populations and *K* = 11 excluding Gambella population. Using these markers, we found strong evidence that the genetic diversity of the lowland bamboo is associated with distinct geographic regions and that isolates of Gambella Region, with their unique genetic origin, are quite different from other bamboos found in the country.

## Graphical abstract

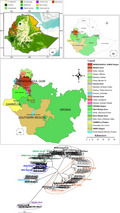

## 1 INTRODUCTION

Bamboo is a member of the grass family, *Poaceae,* and constitutes a single subfamily *Bambusoideae* with 121 genera and 1,662 species (Canavan et al., 2016; Vorontsova, Clark, Dransfield, Govaerts, & Baker, 2016). It is the fastest‐growing plant in the world (up to 100 cm per day; Tao, Fu, & Zhou, 2018) and is one of the most important nontimber forest resources and a potential alternative to wood and wood product (Ekhuemelo, Tembe, & Ugwueze, 2018). Currently, 100 species are commercially cultivated around the world (Li et al., 2020) Bamboo is a multi‐purpose plant, with over 10,000 documented uses and applications. It has rapid regeneration capacity and the possibility of annual harvesting within a few years of planting offers significant advantages over the other forest species (Akinlabi, Anane‐Fenin, & Akwada, 2017; Diver, 2006). Bamboo has also been proven to address many global challenges and contributes to the following United Nations Sustainable Development Goals: SDG 1 (no poverty), 7 (affordable and clean energy), 11 (sustainable and resilient housing), 12 (efficient use of resources), 13 (address climate change), and 15 (life on land) (Bau & Trinh, 2019; Ekhuemelo et al., 2018; Kaushal et al., 2018; Yuen, Fung, & Ziegler, 2017). Due to its potential for water recharge and mitigation soil erosion, bamboo also provides an opportunity for watershed development and restoration of degraded areas (Kaushal et al., 2019, 2020). Furthermore, bamboo is a fodder for livestock and food for humans contributing to ensuring food security (Andriarimalala, Kpomasse, Salgado, Ralisoa, & Durai, 2019; Choudhury, Sahu, & Sharma, 2012; Halvorson, Cassida, Turner, & Belesky, 2011; Mulatu, Bahiru, Kidane, Getahun, & Belay, 2019; Nongdam & Tikendra, 2014). Bamboo has huge economic potential; the global production and local consumption are worth an estimated 60 billion USD, and the international export of the material is valued at USD 2 billion per annum (International Network for Bamboo & Rattan, 2019).

The major species richness of bamboo is found in Asia‐Pacific region, followed by South America. Africa has the fewest number of species is (Bystriakova, Kapos, Lysenko, & Stapleton, 2003), while Europe and Antarctica have no native bamboo species (Zhao et al., 2018). According to the Food and Agriculture Organization's thematic world bamboo resources assessment report and regional remote sensing assessment of bamboo resources in Ethiopia, Kenya, and Uganda, these three countries possess the majority of the bamboo resources in Africa by (Lobovikov et al., 2007; Zhao et al., 2018). Africa is home to 43 species, 40 of which are found mainly in Madagascar, with the remaining three found in mainland Africa (Embaye, 2000). Two indigenous woody bamboo species grow in Ethiopia: the monotypic genus lowland bamboo (*Oxytenanthera abyssinica* [A. Richard] Munro) and the African Alpine Bamboo, or highland bamboo (*Yushania alpina* K. Shumann Lin; synonym: *Arundinaria alpina*, *Oldeania alpine* K. Schumann). These two species are indigenous to Ethiopia and endemic to mainland Africa (Embaye, 2000; Ensermu, Tamrat, Alemayehu, & Gebremedhin, 2000). Ethiopia contributes more than 1.47 million hectares of bamboo coverage (Zhao et al., 2018), which accounts for about 67% of the total bamboo coverage in the continent and 7% of the global coverage (Embaye, 2000). The lowland bamboo (*O. abyssinica*) accounts for 85% of the total national coverage, while the highland bamboo (*A. alpina*) accounts for the remaining 15% (Embaye, 2000; Embaye, Christersson, Ledin, & Weih, 2003; Lobovikov et al., 2007). The lowland bamboo grows in an elevation a range of between 540 to 1,750 m and highland bamboo at a higher elevation above 2,480 m (Zhao et al., 2018).

Assessing the genetic variability of a species within and among different populations is important to devise mechanisms for effective identification, conservation, and multiplication of suitable genetic materials. The genetic diversity of bamboos has not been adequately explored, and relatively limited numbers of molecular finger printing studies have been conducted. The main reason for this exploration of limited genetic diversity is related to the difficulty in assessing the phenotypic variability of clones (Li et al., 2020). Molecular marker techniques such as random amplified polymorphic DNA (RAPD), inter‐simple sequence repeats (ISSR), and amplified fragment length polymorphism (AFLP), simple sequence repeat (SSR), expressed sequence tag derived simple sequence repeat (EST‐SSR), sequence‐related amplified polymorphism (SRAP), restriction fragment length polymorphism (RFLP), and inter‐retrotransposon amplified polymorphism (IRAP) have been used for characterization of some bamboo germplasm (Li et al., 2020; Ma et al., 2013; Nag et al., 2013; Nilkanta, Amom, Tikendra, Rahaman, & Nongdam, 2017; Tian, Yang, Wong, Liu, & Ruan, 2012; Yang, An, Gu, & Tian, 2012) around the world. ISSR molecular markers are widely used for population genetic analysis of different plants, generating more reliable and reproducible bands than RAPD (Nagaoka & Ogihara, 1997; Zhang & Dai, 2010). Use of ISSR is also technically simpler, quicker, and more cost‐effective (Oumer, Yohannes, Kassahun, Abel, & Endashaw, 2015; Tesfaye, Govers, Bekele, & Borsch, 2014) than RFLP, SSR, and AFLP markers, as no previous sequence information is required to generate DNA amplification products (Mukherjee et al., 2010; Tian et al., 2012). ISSR markers are observed to be highly variable within the species and reveal many more polymorphisms since they use longer primers that allow more stringent annealing temperatures (Hillis, Moritz, & Mable, 1996).

Genetic erosion of bamboo and their wild relatives are accelerating at a higher rate because of human activities such as deforestation, wild fire, overexploitation, and the introduction of exotic species without proper research on the potential impact of genetic pollution and problems associated with the transfer of exotic germplasm (Canavan et al., 2016; Tian et al., 2020; Xu et al., 2020). Starting from 2007, Ethiopia has introduced 23 new bamboo species belonging to seven genera (Mulatu, Alemayehu, & Tadesse, 2016). These species are under multiplication at the Holetta and Gurd‐Shola nurseries of the Central Ethiopia Environment and Forest Research Center (CE‐EFRC), Addis Ababa. Overall, it is estimated that about 40 bamboo species are introduced to the country and are being multiplied for planting and/or planted in different geographical locations of the country. Such a massive introduction of exotic species will accelerate the genetic erosion of the native bamboo in the country.

About half of the world`s woody bamboo species are vulnerable to extinction as a result of massive forest destruction (Bystriakova, Kapos, & Lysenko, 2004). In addition to the genetic erosion by exotic species, bamboos in Ethiopia are vulnerable to erosion by human activities. The construction of the Grand Ethiopian Renaissance Dam (GERD) with catchment area covers 172,250 km^2^ (Abtew & Dessu, 2019) is built primarily on major lowland bamboo growing areas of the Metekel Zone of the Benishangul‐Gumuz Region (BGR) and is a prime example of human activities contributing to bamboo deforestation in the country. Moreover, lack and/or gap of knowledge on the plant's biology, genetics, and techniques and technologies for value addition constituting a failure to fulfill the plant's economic potential has also led to unsustainable management of the bamboo plant. The lack of research conducted in Ethiopia, especially on the diversity and systematics of *O. abyssinica* (the species with great ecological and industrial benefit and great coverage in Africa) at DNA level, prompted the commencement of this research. Therefore, in the present study, we used 19 ISSR primers out of 108 initial screenings and 38 final screening of ISSR primers aiming to assess the genetic diversity, population structure, and gene flow analysis of *O. abyssinica* collected from lowland bamboo growing areas in Ethiopia.

## 2 MATERIALS AND METHODS

### 2.1 Plant material collection and sampling strategy

**Figure 1:**
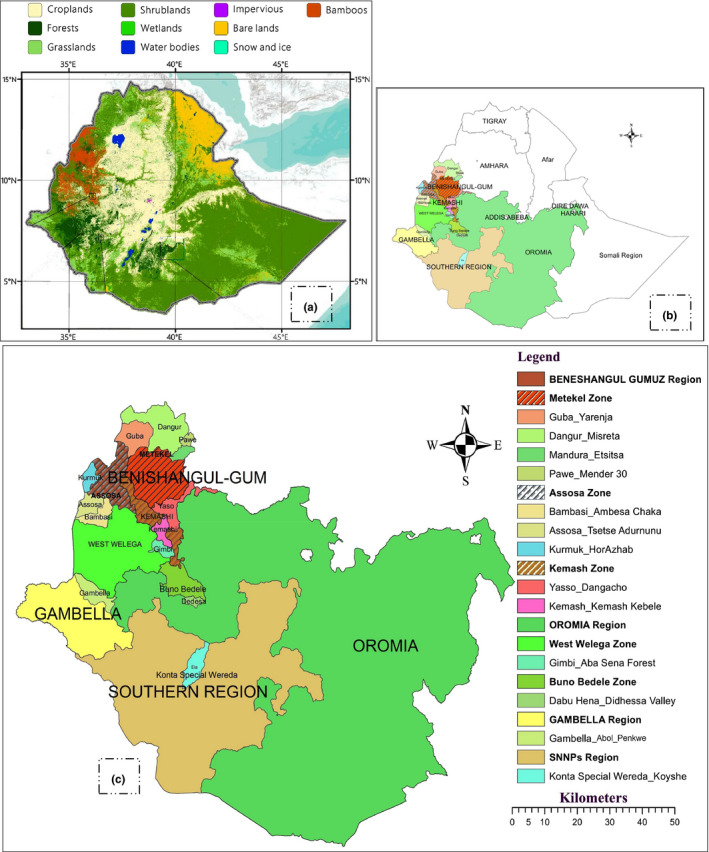
Maps showing sample collection area: (a) Ethiopia's bamboo cover map in the Finer Resolution Observation and Monitoring‐Global Land Cover (FROM‐GLC) classification scheme with other land cover classes; (b) Map of Ethiopia showing sample collection area; and (c) Clipped map showing sample collection area

Young leaves (5–7 in number) from 130 individuals belonging to 13 populations (each population represented by 10 individual bamboo culms) were collected from the natural lowland bamboo (*O. abyssinica*) growing areas of Ethiopia. Since 64.07% of the country's lowland bamboo grows in the BGR (Zhao et al., 2018), nine populations were collected from this region and the remaining four populations were collected from three other bamboo growing regions. GPS data and altitudinal information for each population are presented in Table 1. Maps of collection sites are shown in Figure 1.

**Table 1:** Details of codes, geographic locations and GPS coordinates of bamboo collections

Region	Zone	District	Specific collection site	Sample code	Average GPS reading of the population		Altitude m.a.s.l
					X	Y	
BGR	Metekel	Guba	Yarenja	BGM‐Guba	11°16′13.1″	035°22′15.4″	824
		Dangur	Misreta	BGM‐Dangur	11°18′50.3″	036°14′10.6″	1,240
		Mandura	Etsitsa	BGM‐Mandura	11°09′14.5″	036°19′50.3″	1,039
		Pawe	Mender 30	BGM‐Pawe	11°18′32.5″	036°24′40.2″	1,118
	Assosa	Bambasi	Ambesa Chaka	BGA‐Bambasi	09°53′55.0″	034°40′01.8″	1,518
		Assosa	Tsetse Adurnunu	BGA‐Assosa	10°09′29.9″	034°31′37.1″	1,507
		Kurmuk	HorAzab	BGA‐Kurmuk	10°32′33.7″	034°28′57.9″	1,275
	Kemash	Kemash	Kemash	BGK‐Kemash	09°29′31.4″	035°52′35.2″	1,234
		Yasso	Dangacho	BGK‐Yasso	09°52′27.5″	036°05′32.6″	1,176
Oromia	West Wollega	Gimbi	AbaSena forest	ORWW‐Gimbi	09°01′32.2″	035°59′54.1″	1,407
	BunoBedele	DabuHena	Didhessa valley	ORBB‐Dabu Hena	08°40′21.1″	036°23′32.9″	1,399
Gambella	Gambella	Abol	Penkwe	GGAM‐Abol	08°14′13.1″	034°31′06.2″	435
SNNPs	Konta	Konta	Koyshe	SNNPs‐Konta	06°43′35.6″	036°34′26.8″	958

Abbreviations: BGR, Benishangul‐Gumuz Region; BGM, Benishangul‐Gumuz Region Metekel Zone; BGA, Benishangul‐Gumuz Region Assosa Zone; BGK, Benishangul‐Gumuz Region Kemash Zone; ORWW, Oromia Region West Wollega Zone; ORBB, Oromia Region Buno Bedele Zone; GGAM, Gambella Region Gambella Zone; SNNPs, South Nation's Nationalities and Peoples.

### 2.2 DNA extraction, testing gels, DNA normalization, and primer screening

A total of 130 *O. abyssinica* individuals were used in the study. Five to seven young fresh leaves were preserved in zip‐lock plastic bag with the appropriate amount of silica gel. An amount of 100 mg of silica gel dried leaves was crushed by a mixer and miller (Retsch Mixer Mill MM 400) in the presence of three 3.2 mm diameter stainless steel beads in a 2‐ml sterile centrifuge tube. Genomic DNA was isolated separately using a modified 2% cetyl trimethyl ammonium bromide (CTAB) DNA isolation method at Plant Molecular Biology Laboratory (PMBL) and Plant Genetics Research Laboratory (PGRL) of Addis Ababa University. One milliliter preheated 2% CTAB extraction buffer (2% CTAB, 100 mM Tris‐Base pH 8.0, 25 mM Na_2_‐EDTA, 2 M NaCl, 250 mg/ml PVP and 2% β‐mercaptoethanol) was added to the tube containing crushed leaf powder. The dissolved CTAB mix was incubated for 30 min at 65°C. The tubes were gently inverted every 10 min. Seven hundred microliters of the supernatant (clear liquid only) was transferred to a new sterile centrifuge tube using blue pipette tips, which were cut. Seven hundred microliters of chloroform was added, mixed thoroughly, and centrifuged at 16,000 *g* for 10 min at 26°C. Six hundred microliters of the supernatant was transferred to the new fresh Eppendorf tube, and 60 µl of 3 M sodium acetate (pH 5.2) was added and thoroughly mixed. Six hundred microliters of ice‐cold isopropanol was added and gently mixed by inverting the tubes 3–5 times, and then, the tubes were placed in a refrigerator at −20°C for 2 hr. The mix was centrifuged at 16,000 *g* at 4°C for 5 min to precipitate the DNA. The supernatant was discarded, and the DNA was washed using 1 ml of 70% ethanol by dissolving the pallet completely in the wash buffer and centrifuged at 16,000 *g* for 3 min at 4°C. The wash step was repeated by cold absolute ethanol (1 ml), and the pellet was air‐dried. The pellet was dissolved in 60 µl 0.1X TE (10 mM Tris‐HCl pH 8.0 and 1 mM EDTA pH 8.0) buffer containing RNase. The concentration and quality of DNA were checked by test gel electrophoresis in 1% agarose and measured by using NanoDrop (Thermo Scientific NanoDrop 2000 Spectrophotometer). Each sample was measured, and those with high DNA quality were used for PCR amplification after normalization of each sample to a concentration of 100 ng/μl. Gel documentation was taken by Bio‐Rad Gel Doc™ EZ System Imager.

Thirty‐eight primers (35 designed from the University of British Colombia (UBC) and 3 from previous work) out of 108 ISSR primers (100 from UBC and 8 from the literature) were screened for the initial testing of polymorphism and reproducibility. Nineteen primers were found to be reproducible and polymorphic and used for further ISSR‐PCR work for the study. Among these 19 ISSR primers, ten were dinucleotide, two were tri‐nucleotide, two were tetra‐nucleotide, three were penta‐nucleotide, and two were 5′ anchored primers. Primers were further categorized into 3′ anchored, 5′ anchored, and unanchored based on anchorage property (Table 2).

**Table 2:** Details of ISSR primers used for diversity analysis in this study

No.	Nucleotide Sequence	Primer name	Repeat motifs	Anchorage property	Selected for PCR	Optimized *T*_a_ (^o^C)
1	(AG)_8_T	UBC807	Dinucleotide	3'‐anchored	–	–
2	(AG)_8_C	UBC808	''	''	–	–
3	(GA)_8_T	UBC810	''	''	√	42
4	(GA)_8_A	UBC812	''	''	√	42
5	(CT)_8_T	UBC813	''	''	–	–
6	(CT)_8_G	UBC815	''	''	√	42
7	(TC)_8_C	UBC823	''	''	–	–
8	(TC)_8_G	UBC824	''	''	√	43
9	(AC)_8_T	UBC825	''	''	–	–
10	(AG)_8_YT	UBC834	''	''	√	43
11	(AG)_8_YC	UBC835	''	''	√	45
12	(GA)_8_YT	UBC840	''	''	√	42
13	(GA)_8_YC	UBC841	''	''	√	43
14	(GA)_8_YG	UBC842	''	''	–	–
15	(CT)_8_RC	UBC844	''	''	√	43
16	(CT)_8_RG	UBC845	''	''	–	–
17	(CA)_8_RG	UBC848	''	''	√	47
18	(GT)_8_YG	UBC851	''	''	–	–
19	(TC)_8_RT	UBC853	''	''	–	–
20	(TC)_8_RG	UBC854	''	''	–	–
21	(AC)_8_YG	UBC857	''	''	–	–
22	(ACC)_6_	UBC861	Tri	Unanchored	√	55
23	(AGC)_6_	UBC862	''	''	–	–
24	(AGT)_6_	UBC863	''	''	–	–
25	(ATG)_6_	UBC864	''	''	√	39
26	(GATA)_4_	UBC872	Tetra	“	–	–
27	(GACA)_4_	UBC873	''	''	√	42
28	(CCCT)_4_	UBC874	''	''	–	–
29	(CTAG)_4_	UBC875	''	''	–	–
30	(GATA)_2_(GACA)_2_	UBC876	''	''	√	39
31	(CTTCA)_3_	UBC879	Penta	''	–	–
32	(GGAGA)_3_	UBC880	''	''	√	45
33	(GGGTG)_3_	UBC881	''	''	√	47
34	BDB(CA)_7_	UBC888	5′‐anchored	5′‐anchored	√	47
35	DBD(AC)_7_	UBC889	''	''	√	45
36	(AGG)_6_	ISSR_1	Tri	Unanchored	–	–
37	(ACTG)_4_	ISSR_2	Tetra	''	–	–
38	(GACAC)_4_	ISSR_3	Penta	''	√	55

Note:

Y = C or T → (Pyrimidine); R = A or G → (Purine); B = C, G, or T → (not A); D = A, G, or T → (not C).

### 2.3 ISSR‐PCR amplification and gel electrophoresis

Each DNA amplification reaction was performed in a final volume of 10 µl containing 5.5 µl of 2x Taq plus Master Mix (containing Taq DNA polymerase, dNTPs, MgCl_2_, PCR buffer, PCR reaction enhancer, stabilizer, and a blue tracer dye), 3.5 µl of ddH_2_O, 0.5 µl of ISSR primer (0.2 pmol/µl), and 0.5 µl of normalized genomic DNA (100 ng). The blue dye and a stabilizer of 2x Taq plus Master Mix helped to directly load the final products onto a gel for analysis. The thermal profile included pre‐PCR denaturation at 94°C for 4 min followed by 35 cycles of denaturing at 94°C for 30 s, annealing for 30 s at the optimized temperature (details are given for each respective primer given in Table 2), extension at 72°C for 1 min, and a final extension at 72°C for 10 min. The PCR products were stored at 4°C until loading on agarose gel electrophoresis. Five microliters of the ISSR‐amplification product of each sample was loaded on 1.67% agarose gel and in 0.5x TBE buffer at a constant voltage of 100 V for 1:30 – 2 hr. The agarose gel was stained with 3.0 µl ethidium bromide after dissolving the agarose powder in 0.5% TBE buffer. The ISSR bands were visualized and photographed under Bio‐Rad Gel Doc™ EZ System Imager that was connected to a PC with Image Lab software and stored for later data scoring. To estimate the molecular sizes of the resolved fragments, a 100 bp DNA marker was used.

### 2.4 Scoring of bands

ISSR bands were scored manually. According to the weight of the DNA ladder (100 bp), the same weight bands were marked as a line. The bands that were clearly visible and repeatable on the electrophoresis map were marked as "1," the absence of a band at the same site was marked as "0" and ambiguous bands were considered as a missing data and marked as "?". Intensity variations among fragments having approximately the same molecular size were not considered although in some cases intensity differences of the bands were observed. A binary data matrix was compiled with individuals in the column and the ISSR markers in the row for each primer set and vice‐versa according to the requirements of the software. Each amplified fragment was named by the code of the primers across the row and/or column followed by the Arabic numbers starting from the fragment having high molecular weight to the fragments with low‐molecular weight. Both the total number of bands amplified by each primer and the number of polymorphic bands were calculated.

### 2.5 Data analysis

#### 2.5.1 Band pattern frequency, markers efficiency, and gene diversity analysis

On the basis of the recorded band profiles, different software was employed for data analysis. POPGENE version1.32 (Yeh & Boyle, 1999) and GenAlEx (genetic analysis in excel) version 6.5 (Peakall & Smouse, 2012) were used to calculate genetic diversity for each population as the number of polymorphic loci, percent polymorphism, gene diversity, and Shannon diversity index. GenAlEx6.5 was also used to calculate band patterns on its frequency and polymorphism. The polymorphism information content (PIC), marker index (MI), expected heterozygosity (*H*), and discriminating power (*D*) were inferred via iMEC (https://irscope.shinyapps.io/iMEC/) online.

#### 2.5.2 Proportion of genetic variability within and among populations

Analysis of molecular variance (AMOVA) was used to calculate the *F*‐statistics that were used to estimate the proportion of genetic variability found among populations (*F*_ST_), among populations within groups (*F*_SC_), and among groups (*F*_CT_) using Areliquin version 3.01 (Excoffier & Lischer,  2006) and GenAlEx6.503 (Peakall & Smouse, 2012). The genetic similarity matrix among the 130 individual samples of *O. abyssinica* was calculated in all pair‐wise comparisons following Jaccard's similarity coefficients.

#### 2.5.3 Clustering and admixture analysis

NTSYS‐pc version 2.02 (Rohlf, 2000) was used to generate the unweighted pair group method with arithmetic mean (UPGMA) phenogram, allowing for a sequential, agglomerative, hierarchical, and nested (SAHN) cluster analysis using the similarity matrix and compare the individual genotypes. The neighbor‐joining (NJ) method (Saitou & Nei, 1987; Studier & Keppler, 1988) was used to compare individual genotypes and evaluate patterns of genotype clustering using Free Tree 0.9.1.50 Software (Pavlicek, Hrda, & Flegr, 1999) and TreeView (Page, 1996). Patterns of genetic variation among individual samples were also further examined in three dimensions with the help of principal coordinate analysis (PCoA) on the basis of Jaccard's coefficients of similarities, which was calculated using PAST software version1.18 (Hammer, Harper, & Ryan, 2001). The first three axes were later used to construct the scatter plot with STATISTICA version 12.0 software's (StatSoft & Inc., 2010) and XLSTAT 2014.5.03.

A Bayesian model‐based clustering algorithm in STRUCTURE ver. 2.3.4 (Falush, Stephens, & Pritchard, 2003; Pritchard, Stephens, & Donnelly, 2000) was applied to infer the pattern of population structure and detection of admixture. To determine the most likely number of populations (*K*), a burn‐in period of 50,000 was used in each run, and data were collected over 500,000 Markov Chain Monte Carlo (MCMC) replications for *K* = 1 to *K* = 13 and *K* = 1 to *K* = 12 (excluding samples from Gambella Region) using 20 iterations for each *K*. Each test takes more than 300 hr. for analysis using a PC with 8GB RAM and a core i7 processor. The optimum *K* value was predicted following the simulation method of Evanno, Regnaut, & Goudet, 2005 using the web‐based STRUCTURE HARVESTER ver. 0.6.94 (Earl, 2012). A bar plot for the optimum K was determined using Clumpak (Cluster Markov Packager Across K) beta version (Kopelman, Mayzel, Jakobsson, Rosenberg, & Mayrose, 2015).

## 3 RESULTS

### 3.1 ISSR marker banding patterns

**Figure 2:**
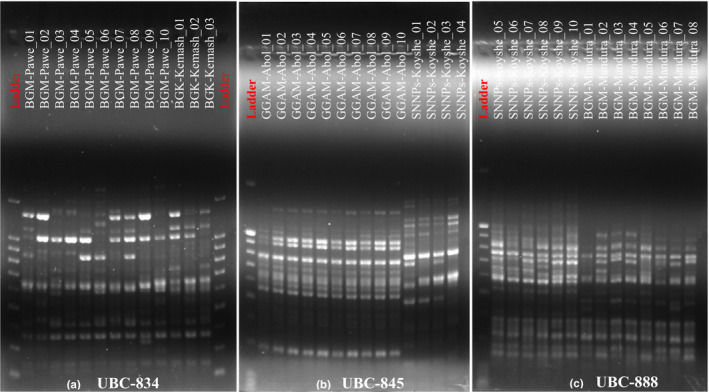
A representative of ISSR‐PCR electrophoresis profile of 13 populations of *O. abyssinica* using (a) UBC‐834, (b) UBC‐845, and (c) UBC‐888

Based on the results from gel pictures taken for each primer, the pattern of DNA amplification obtained was clear and reproducible (Figure 2) even though scoring of bands and interpretation of certain gel pictures were challenging. The size of the band generated ranged from 100 to 1,700 bp (Table 3). The number of bands produced by each primer varied from 13 bands for UBC‐844 to 24 for UBC‐815. Among 348 scored bands, 294 (84.48%) of fragments showed polymorphisms. Based on the repeat motif property of primers, dinucleotides produced 185 scorable bands, tri‐nucleotides 32, tetra‐nucleotides 39, and penta‐nucleotides produced 56 scorable bands. Based on the anchorage property of primers, anchored primers produced 221 bands and unanchored primers produced 127 bands. 3′ anchored (are also dinucleotides) produced 185 bands, whereas 5′ anchored primers produced 56 bands (Table 3).

**Table 3:** Band polymorphism produced by 19 ISSR primers from 13 populations of *O. abyssinica* along with molecular size ranges in base pairs (bp)

Primers	Individual primers						Molecular size range in bp
	NSB	NPL	PPL (%)	*H* ± *SD*	*I* ± *SD*	*N*_m_	
UBC‐810	18	16	88.89	0.2130 ± 0.2037	0.3322 ± 0.2632	2.1307	1,400–100
UBC‐812	19	19	100	0.3747 ± 0.1784	0.5428 ± 0.2197	2.9205	1,300–300
UBC‐815	24	24	100	0.3318 ± 0.1638	0.4973 ± 0.1992	2.2082	1,400–200
UBC‐824	16	15	93.75	0.3149 ± 0.1563	0.4749 ± 0.2082	1.5946	1,600–300
UBC‐834	22	22	100	0.3712 ± 0.1173	0.5506 ± 0.1389	1.4004	1,700–200
UBC‐835	23	22	95.65	0.2776 ± 0.1623	0.4307 ± 0.2046	1.1409	1,600–100
UBC‐840	16	15	93.75	0.2826 ± 0.2044	0.4242 ± 0.2629	2.9005	1,500–150
UBC‐841	16	14	87.50	0.3050 ± 0.2012	0.4500 ± 0.2665	2.5796	1,100–100
UBC‐844	13	12	92.31	0.2314 ± 0.1714	0.3678 ± 0.2243	1.2566	950–350
UBC‐848	18	15	83.33	0.1451 ± 0.1633	0.2546 ± 0.2047	0.3496	950–150
UBC‐861	14	12	85.71	0.2530 ± 0.1835	0.3905 ± 0.2437	0.3456	1,300–300
UBC‐864	18	13	72.22	0.2775 ± 0.1983	0.4087 ± 0.2832	1.8663	1,400–400
UBC‐873	21	16	76.19	0.2603 ± 0.2106	0.3857 ± 0.2884	2.5901	900–200
UBC‐876	18	13	72.22	0.2520 ± 0.2214	0.3711 ± 0.3012	2.2620	1,100–300
UBC‐880	21	19	90.48	0.3311 ± 0.1962	0.4844 ± 0.2537	1.1703	1,100–200
UBC‐881	19	13	68.42	0.1913 ± 0.2157	0.2899 ± 0.2912	1.9785	1,100–200
UBC‐888	22	12	54.55	0.1542 ± 0.2121	0.2316 ± 0.2911	3.2746	950–300
UBC‐889	14	9	64.29	0.2619 ± 0.2297	0.3778 ± 0.3197	2.0015	900–200
(GACAC)4	16	13	81.25	0.2594 ± 0.2081	0.3892 ± 0.2775	0.8541	1,200–300
Average	**18.32**	**15.58**	**84.48**	**0.2702 ± 0.1945**	**0.4061 ± 0.2595**	**1.5474**	
All primers	**348**	**294**					
Based on repeat motif							
Dinucleotides	185	174	94.05	0.2894 ± 0.1780	0.4389 ± 0.2288	1.6598	
Tri‐nucleotides	32	25	78.12	0.2668 ± 0.1893	0.4007 ± 0.2626	0.8552	
Tetra‐nucleotides	39	29	74.36	0.2565 ± 0.2128	0.3789 ± 0.2906	2.4322	
Penta‐nucleotides	56	45	80.36	0.2632 ± 0.2112	0.3912 ± 0.2811	1.1952	
Based on anchorage property							
3′ anchored	185	174	94.05	0.2894 ± 0.1780	0.4389 ± 0.2288	1.6598	
5′ anchored	36	21	58.33	0.1961 ± 0.2223	0.2884 ± 0.3067	2.4821	
3′ + 5′ anchored	221	195	88.24	0.2742 ± 0.1885	0.4144 ± 0.2487	1.7315	
Unanchored	127	99	77.95	0.2620 ± 0.2048	0.3898 ± 0.2775	1.3081	

Note

NSB ‐ number of scorable bands, NPL ‐ number of polymorphic loci, PP ‐ percent polymorphism, *H* ‐ genetic diversity, *I* ‐ Shanon's information index and *N*_m_ ‐ estimate of gene flow. (**Remark)**: all dinucleotides are 3′ anchored.

### 3.2 Level of polymorphism of Ethiopian lowland bamboo

#### 3.2.1 ISSR primers on genetic polymorphism of Ethiopian lowland bamboo

The highest number of scorable bands (NSB) was produced by primers UBC‐815 (24), UBC‐835 (23), UBC‐834 (22), and UBC‐888 (22) and the lowest NSB were produced by primers UBC‐844 (13) and UBC‐889 (14). The number of polymorphic loci (NPL) ranges from 24 (UBC‐815) to 9 (UBC‐889). Percentage of polymorphic loci (PPL) was 100% for primers UBC‐812, UBC‐815, and UBC‐834, the lowest PPL were exhibited by primers UBC‐888 (54.55%), and UBC‐889 (64.29%) and the average PPL for all primers were 84.48%. Based on the repeat motifs and anchorage property of primers, dinucleotide repeat motifs showed higher PPL (94.05) than tri‐ (78.12), tetra‐ (74.36), and penta‐nucleotide (80.36) repeat motifs. 3′ anchored primers showed higher PPL (94.05) than 5′ anchored (58.33). Anchored primers show higher PPL (88.24) than unanchored primers (77.95).

The lowest heterozygosity (*H*) value (0.1451 ± 0.1633) was shown by primer UBC‐848, and the highest (0.3747 ± 0.1784) was exhibited by primer UBC‐812. Again, dinucleotide repeats showed the highest heterozygosity (0.2894 ± 0.1780). Similarly, the maximum and minimum Shannon's information index (*I*) was 0.5506 ± 0.1389 by UBC‐834 and 0.5428 ± 0.2197 by UBC‐812. The highest estimate of gene flow (*N*_m_) was observed in UBC‐888 (3.2746) and least *N*_m_ was observed in UBC‐861 (0.3456). The average *N*_m_ for overall primers was 1.5474. Tetra‐nucleotide (2.4322) and 5′ anchored (2.4821) primers showed the highest *N*_m_ values (Table 3).

#### 3.2.2 Analysis of markers efficiency

The highest expected heterozygosity (*H*), polymorphism information content (PIC), and discriminating power (*D*) values were shown by anchored primers in general and 3′ anchored and dinucleotide repeats (UBC‐834 and UBC‐835) in particular. The *H*, PIC, MI, and *D* values for anchored primers were 0.503661, 0.470489, 0.503661, and 0.455827, respectively; 3′ anchored and dinucleotide repeats were 0.520932, 0.459651, 0.520932, and 0.698063; UBC‐834 were 0.732223, 0.507292, 0.732223, and 0.438911; and UBC‐835 were 0.686454, 0.508061, 0.686454, and 0.323554. The lowest *H*, PIC, and *D* values were observed in tri‐nucleotides and 5′ anchored primers. Again, dinucleotide and anchored primers showed higher *D* values than other repeat motif types and unanchored primers (Table 4).

**Table 4:** Polymorphism statistics calculated using iMEC for the *O. abyssinica* populations based on 19 ISSR primers data set

ISSR Primer/Marker	*H*	PIC	MI	*D*
UBC‐810	0.465799	0.447393	0.465799	0.863768
UBC‐812	0.499475	0.43114	0.499475	0.766033
UBC‐815	0.515089	0.46009	0.515089	0.844063
UBC‐824	0.482959	0.439253	0.482959	0.833903
UBC‐834	0.732223	0.507292	0.732223	0.920051
UBC‐835	0.686454	0.508061	0.686454	0.896514
UBC‐840	0.485022	0.438254	0.485022	0.829166
UBC‐841	0.499998	0.430879	0.499998	0.751081
UBC‐844	0.436556	0.460587	0.436556	0.323554
UBC‐848	0.40574	0.473565	0.40574	0.438911
Dinucleotides (3′ anchored)	**0.520932**	**0.459651**	**0.520932**	**0.746704**
UBC‐861	0.477973	0.441648	0.477973	0.357648
UBC‐864	0.483738	0.438876	0.483738	0.651802
Tri‐nucleotides (Unanchored)	**0.480856**	**0.440262**	**0.480856**	**0.504725**
UBC‐873	0.495109	0.433311	0.495109	0.698195
UBC‐876	0.537861	0.460926	0.537861	0.696486
Tetra‐nucleotides (Unanchored)	**0.516485**	**0.447119**	**0.516485**	**0.697341**
UBC‐880	0.499527	0.431114	0.499527	0.269529
UBC‐881	0.494796	0.433466	0.494796	0.253031
(GACAC)4	0.499963	0.430896	0.499963	0.745775
Penta‐nucleotides (Unanchored)	**0.498095**	**0.431825**	**0.498095**	**0.422778**
UBC‐888	0.482824	0.474689	0.482824	0.157652
UBC‐889	0.489958	0.487964	0.489958	0.73447
5′ anchored	**0.486391**	**0.481327**	**0.486391**	**0.446061**
Overall	**0.509003**	**0.454179**	**0.509003**	**0.633244**
Summary based on primers repeat motifs				
Dinucleotides	**0.520932**	**0.459651**	**0.520932**	**0.746704**
Tri‐nucleotides	**0.480856**	**0.440262**	**0.480856**	**0.504725**
Tetra‐nucleotides	**0.516485**	**0.447119**	**0.516485**	**0.697341**
Penta‐nucleotides	**0.498095**	**0.431825**	**0.498095**	**0.422778**
Summary based on primers' anchorage property				
3′ anchored	**0.520932**	**0.459651**	**0.520932**	**0.746704**
5′ anchored	**0.486391**	**0.481327**	**0.486391**	**0.446061**
3′ + 5′ anchored	**0.503661**	**0.470489**	**0.503661**	**0.596383**
Unanchored	**0.49885**	**0.437525**	**0.49885**	**0.592655**

Abbreviations: *D*, discriminating power; *E*, effective multiplex ratio; *H*, expected heterozygosity; Hav, mean heterozygosity; MI, marker index; PIC, polymorphism information content; R, resolving power.

#### 3.2.3 Band pattern and heterozygosity

The highest number of band patterns was observed in the Konta population (227) followed by Guba and Pawe populations (219) each. The highest number of private bands was observed in Gambella populations (69), 6 in Konta, and 1 in the Pawe population. There were no private bands in the other populations. A majority of the bands was obtained from the number of different bands with a frequency of ≥5% (NDBF ≥ 5%). Mean of expected heterozygosity (He**)** and mean of unbiased expected heterozygosity (uHe) along with standard errors showed the highest values in the Guba (0.219 ± 0.013, 0.231 ± 0.013) and Dabu Hena populations (0.218 ± 0.013, 0.229 ± 0.014) (Table 5).

**Table 5:** Band patterns observed across *O. abyssinica* populations in Ethiopia using nineteen ISSR primers

Population	NDB	NDBF ≥5%	NPB	NLCB (≤25%)	NLCB (≤50%)	Mean *H*e ± *SE*	Mean u*H*e ± *SE*
Gambella‐Gambella							
Abol	215	215	66	24	35	0.198 ± 0.013	0.208 ± 0.013
SNNPs‐Konta Special Woreda							
Konta	227	227	6	16	28	0.210 ± 0.013	0.221 ± 0.014
Benishangul‐Gumuz‐Metekel Zone							
Mandura	195	195	0	6	13	0.184 ± 0.012	0.194 ± 0.013
Dangur	211	211	0	2	14	0.213 ± 0.013	0.225 ± 0.014
Guba	219	219	0	6	19	0.219 ± 0.013	0.231 ± 0.013
Pawe	219	219	1	10	21	0.208 ± 0.013	0.219 ± 0.013
Benishangul‐Gumuz‐Kemash Zone							
Kemash	210	210	0	2	10	0.213 ± 0.013	0.224 ± 0.014
Yasso	212	212	0	3	11	0.193 ± 0.013	0.204 ± 0.013
Benishangul‐Gumuz‐Assosa Zone							
Assosa	205	205	0	2	13	0.204 ± 0.013	0.215 ± 0.014
Bambasi	210	210	0	4	17	0.205 ± 0.013	0.216 ± 0.013
Kurmuk	200	200	0	2	10	0.196 ± 0.013	0.207 ± 0.013
Oromia‐West Wellega Zone							
Gimbi	210	210	0	2	11	0.203 ± 0.013	0.214 ± 0.014
Oromia‐Buno Bedele Zone							
Dabu Hena	214	214	0	5	16	0.218 ± 0.013	0.229 ± 0.014

Note

NDB = No. of different bands, NDBF ≥ 5% = No. of different bands with a frequency ≥ 5%, NPB = No. of bands unique to a single population, NLCB (≤25%) = No. of locally common bands (Freq. ≥5%) found in 25% or fewer populations, NLCB(≤50%) = No. of locally common bands (Freq. ≥ 5%) found in 50% or fewer populations, He = Expected heterozygosity = 2 * *p* * *q*, uHe = Unbiased expected heterozygosity = (2N/(2N − 1)) * He Where for Diploid Binary data and assuming Hardy‐Weinberg Equilibrium, *q* = (1 ‐ Band Freq.)^0.5 and *p* = 1 − *q*.

### 3.3 Genetic polymorphism and Shanon's information index

Among the thirteen populations, the Guba population was identified as having the highest percent polymorphism (47.13%) followed by Dabu Hena (45.40%) and Bambasi (44.83%). Populations from Mandura (40.52%), Gambella, and Yasso (41.09%), on the other hand, were observed to have the lowest percent polymorphism. High genetic variation at the species level was observed in the present investigation with 84.48% PPL. The values for Nei's genetic diversity (*H*), Shannon's diversity index (*I*), observed number of alleles (Na), and effective number of alleles (Ne) along with standard deviation (*SD*) at species levels were also 0.2702 ± 0.1945, 0.4061 ± 0.2595, 1.8448 ± 0.3626, and 1.4744 ± 0.3960, respectively, showing a relatively high level of genetic diversity (Table 6). However, genetic differentiation at the population level was relatively low when compared to genetic variation evident at the species level. This was proven by relatively moderate PPL recorded in the range of 40.52% to 47.13% and averaging 43.41%. Guba showed higher *H* value (0.2193 ± 0.2383) followed by Dabu Hena or Didhesa valley population (0.2165 ± 0.2407).The Mandura, Abol, and Yasso populations were observed to be the least diverse, with gene diversity values of 0.1842 ± 0.2285, 0.1978 ± 0.2390, and 0.1922 ± 0.2342, respectively. The same diversity patterns were also observed for *I*, Na, and Ne, whereby Guba, Dabu Hena, and Bambasi populations showed the highest and Mandura, Yasso, and Abol populations showed the least. In general, Metekel Zone of BGR showed the highest NPL, PPL, *H*, *I*, Na, and Ne even though the Buno Bedele Zone of Oromia Region showed the highest value by single population. Polymorphism within the population was found to be lower than 50% for all populations. Additionally, the total genetic diversity (Ht) was 0.2708 ± 0.0379, the genetic diversity within populations (Hs) was 0.2047 ± 0.0327, the coefficient of gene differentiation (*G*_st_) was = 0.2442, and estimate of gene flow among populations (*N*_m_) was = 1.5474) (Table 6).

**Table 6:** Genetic diversity within populations and genetic differentiation parameters of 13 populations of *O. abyssinica*

Populations	With all primers					
	NPL	PPL (%)	(*H* ± *SD*)	(*I* ± *SD*)	Na	Ne
Gambela						
GGAM‐Abol	143	41.09	0.1978 ± 0.2390	0.2767 ± 0.3336	1.4109 ± 0.4927	1.3857 ± 0.4686
SNNPs						
SNNPs‐Konta	153	43.97	0.2102 ± 0.2408	0.2942 ± 0.3359	1.4397 ± 0.4971	1.4097 ± 0.4730
B‐Gumuz‐Metekel Zone						
BGM‐Mandura	141	40.52	0.1842 ± 0.2285	0.2611 ± 0.3214	1.4052 ± 0.4916	1.3484 ± 0.4408
BGM‐Dangur	155	44.54	0.2112 ± 0.2400	0.2962 ± 0.3349	1.4454 ± 0.4977	1.4102 ± 0.3349
BGM‐Guba	164	47.13	0.2193 ± 0.2383	0.3088 ± 0.3330	1.4713 ± 0.4999	1.4224 ± 0.4661
BGM‐Pawe	155	44.54	0.2078 ± 0.2366	0.2927 ± 0.3310	1.4454 ± 0.4977	1.3996 ± 0.4622
Zone Mean	**153.75**	**44.1825**	**0.2056 ± 0.2358**	**0.2897 ± 0.3301**	**1.4418 ± 0.4967**	**1.3952 ± 0.4260**
B‐Gumuz‐Kemash Zone						
BGK‐Kemash	155	44.54	0.2129 ± 0.2411	0.2981 ± 0.3363	1.4454 ± 0.4977	1.4150 ± 0.4735
BGK‐Yasso	143	41.09	0.1922 ± 0.2342	0.2705 ± 0.3281	1.4109 ± 0.4927	1.3690 ± 0.4543
Zone Mean	**149**	**42.815**	**0.2025 ± 0.2376**	**0.2843 ± 0.3322**	**1.4282 ± 0.4952**	**1.3920 ± 0.4639**
B‐Gumuz‐Assosa Zone						
BGA‐Assosa	147	42.24	0.2040 ± 0.2407	0.2851 ± 0.3356	1.4224 ± 0.4947	1.3987 ± 0.4730
BGA‐Bambasi	156	44.83	0.2056 ± 0.2353	0.2902 ± 0.3292	1.4483 ± 0.4980	1.3937 ± 0.4583
BGA‐Kurmuk	146	41.95	0.1962 ± 0.2353	0.2761 ± 0.3293	1.4195 ± 0.4942	1.3773 ± 0.4571
Zone Mean	**149.67**	**43.01**	**0.2019 ± 0.2371**	**0.2838 ± 0.3314**	**1.4301 ± 0.4956**	**1.3899 ± 0.4628**
Oromia‐W/Wellega						
ORWW‐Gimbi	148	42.53	0.2023 ± 0.2399	0.2842 ± 0.3344	1.4253 ± 0.4951	1.3961 ± 0.4717
Oromia‐Buno‐Bedele						
ORBB‐Dabu Hena	158	45.40	0.2165 ± 0.2407	0.3035 ± 0.3360	1.4540 ± 0.4986	1.4207 ± 0.4721
Mean of Populations	**151.0769**	**43.4131**	**0.2046 ± 0.2377**	**0.2875 ± 0.3322**	**1.4341 ± 0.4960**	**1.3959 ± 0.4543**
Overall for species	**294**	**84.48**	**0.2702 ± 0.1945**	**0.4061 ± 0.2595**	**1.8448 ± 0.3626**	**1.4744 ± 0.3960**
Summary of Genic Variation Statistics for all Loci Total and (Mean ± *SD*)	*H*_t_	0.2708 ± 0.0379				
	*H*_s_	0.2047 ± 0.0327				
	*G*_st_	0.2442				
	*N*_m_	1.5474				

Note

NPL ‐ Number of polymorphic loci; PPL ‐ percentage of polymorphic loci; *H* ‐ Nei's gene diversity; *I* ‐ Shannon's information index; Na ‐ observed number of alleles; Ne ‐ effective number of alleles; *H*_t_ ‐ total genetic diversity; *H*_s_ ‐ genetic diversity within populations; *G*_st_ ‐ the coefficient of gene differentiation; and *N*_m_ ‐ estimate of gene flow among populations.

### 3.4 Analysis of molecular variance

Analysis of molecular variance (AMOVA) was carried out in two phases; the first phase focused on the entire population's overall loci by considering them as one geographic region and the second phase separated populations into seven groups based on administrative Zones. The analysis was carried out by computation of the distance between “haplotypes,” each individual's data pattern as one “haplotype” and computing variance components for each level (Excoffier & Lischer, 2006).

Partitioning of genetic diversity by AMOVA (Table 7) using grouped populations revealed that out of total genetic diversity, most of the ISSR diversity was distributed within the populations (61.05%); remaining diversity was distributed among groups (31.80%) and among populations within groups (7.15%), with *F*_ST_ = 0.38949, *F*_SC_ = 0.10486, and *F*_CT_ = 0.31797. Similarly, partitioning of genetic diversity without grouping revealed that out of total genetic diversity, most of the ISSR diversity was due to differences between individual plants within the populations (63.65%), while the remaining diversity was due to differences among populations (36.35%) with a *F*_ST_ value of 0.36354. In both cases, the analysis of molecular variance revealed the same patterns of genetic diversity and support the larger genetic diversity found within the populations rather than among the populations, and are similar to Shannon's diversity index.

**Table 7:** AMOVA for the entire 13 populations and seven administrative Zonal groups of *O. abyssinica* in Ethiopia

	Source of variation	*df*	Sum of squares	Variance components	Percentage of variation
With seven Zonal grouping	Among Groups	6	2,124.524	16.38900 Va	31.80
	Among Populations within Groups	6	409.983	3.68630 Vb	7.15
	Within Populations	117	3,681.700	31.46752 Vc	61.05
	Total	129	6,216.208	51.54282	
Fixation Indices	*F*_SC_ = 0.10486	*F*_ST_ = 0.38949		*F*_CT_ = 0.31797	
Entire Ethiopian populations	Among Populations	12	2,534.508	17.97415 Va	36.35
	Within Populations	117	3,681.700	31.46752 Vb	63.65
	Total	129	6,216.208	49.44167	
Fixation Index	*F*_ST_ = 0.36354				

### 3.5 Cluster analysis

Intrageographical cluster analysis of UPGMA (Unweighted Pair Group Methods using arithmetic Averages) and NJ (Neighbor‐Joining) was computed for all individuals and populations of *O. abyssinica*. The UPGMA dendrogram resulting from a SAHN clustering analysis and NJ analysis on the basis of Jaccard's coefficients of similarity was constructed. The Jaccard's coefficient of similarity was obtained after pair‐wise comparisons were performed using binary character matrices (of the presence and absence of bands) that were produced from amplified fragments.

**Figure 3:**
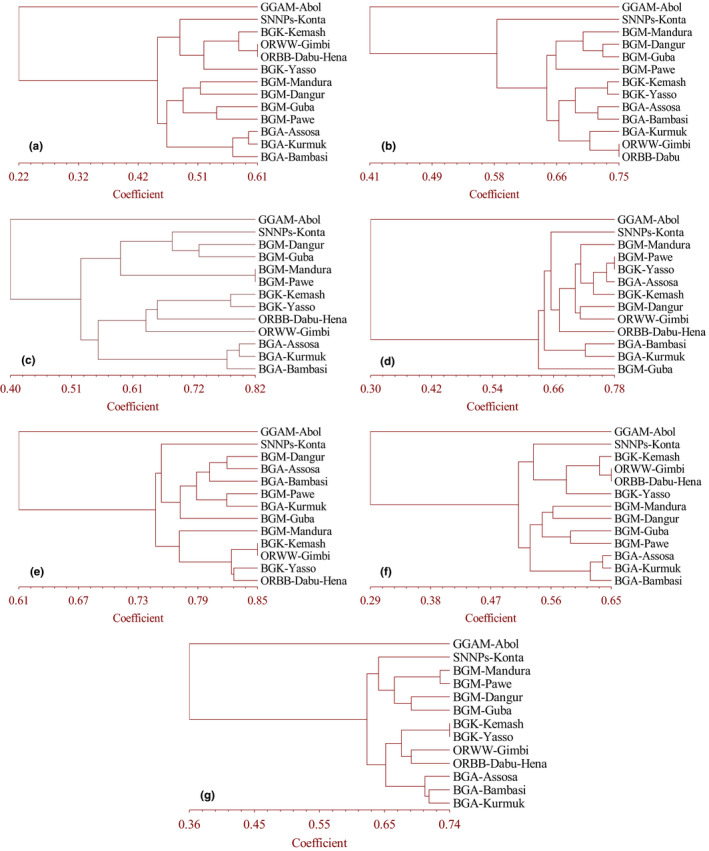
Dendrogram depicting clustering patterns for thirteen (13) populations of *O. abyssinica* based on Jaccard's similarity coefficient. (a) Dinucleotide also 3′ anchored, (b) Tri‐nucleotide, (c) Tetra‐nucleotide, (d) Penta‐nucleotide, (e) 5′ anchored, (f) 3′ and 5′ anchored and (g) Unanchored ISSR primers

**Figure 4:**
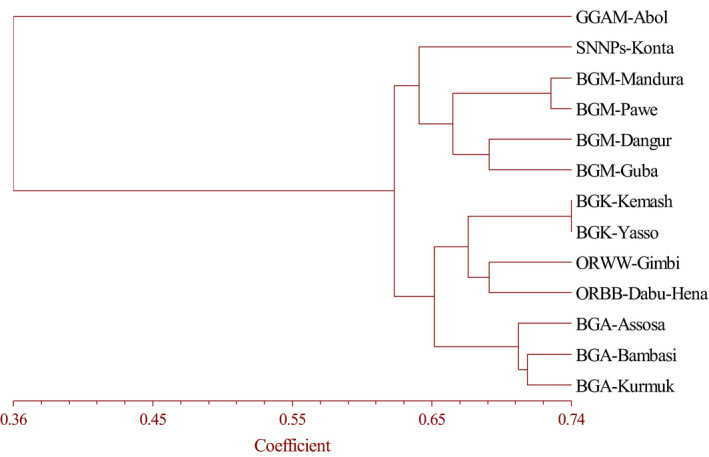
UPGMA‐based dendrogram for thirteen *O. abyssinica* populations based on Jaccard's similarity coefficient using 19 ISSR primers

**Figure 5:**
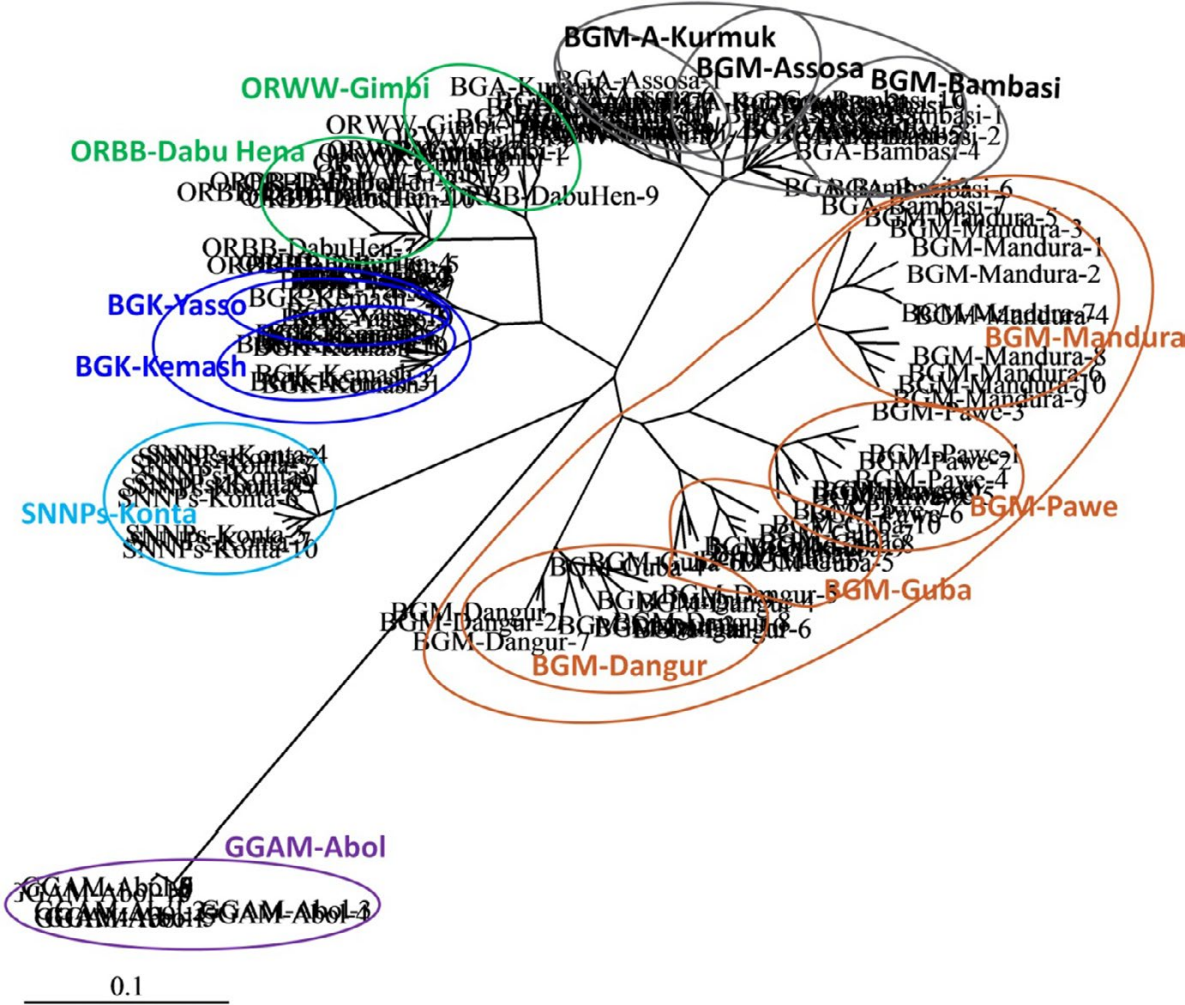
NJ analysis of 130 individuals based on Jaccard's similarity coefficient. Samples encircled inside the bigger circles represent a single population and bigger circle represent Zonal samples

Most primer combinations in terms of nucleotide repeats and anchorage property showed almost similar clusters like the overall assessment. However, tri‐nucleotide repeat ISSR primer mixes Kurmuk samples of BGR with those of the Oromia Region, areas that are distant from one another. 5′ anchored primers, on the other hand, mix samples from the Kemash Zones of the BGR samples with those of Oromia Region. The poorest tree topology was observed by penta‐nucleotide primers that mix Assosa, Pawe, and Yasso populations in the same cluster while they were geographically very distant and even in three separate administrative zones of BGR (Figure 3a‐g). Generally, dinucleotides also 3′ anchored primers and anchored primers showed better clustering results (Figure 3a). Moreover, the UPGMA (Figure 4) and NJ (Figure 5) clustering methods of 130 individuals of *O. abyssinica* all 19 ISSR primers produced exactly the same tree topology according to their geographic distance and location.

### 3.6 Principal coordinate (PCoA) analysis

**Figure 6:**
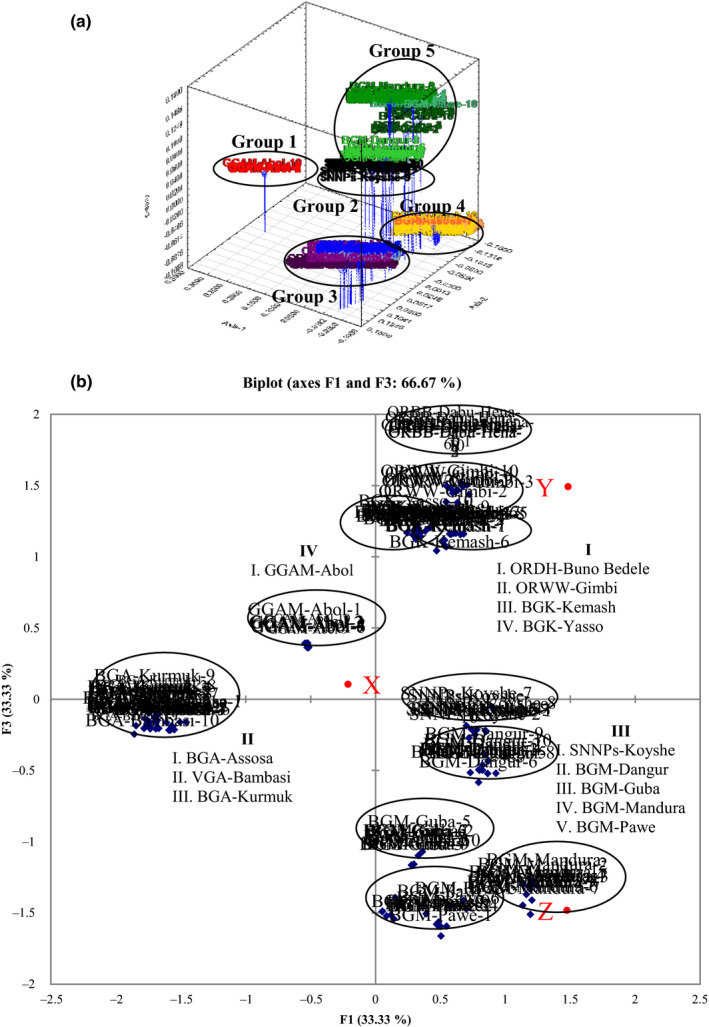
Three‐dimensional representation of a principal coordinate analysis of genetic relationships among 130 individuals of 13 populations of *O. abyssinica* inferred from a similarity matrix using (a) the Jaccard's index at STATISTICA and (b) Pearson PCoA method using XLSTAT

The data obtained from 19 ISSR primers were used in PCoA analysis using Jaccard's coefficients of similarity for grouping of individuals and clustering of *O. abyssinica* using three coordinates (Figure 6a,b). The analysis was carried out using STATISTICA version 12.0 and XLSTAT 2014.5.03 software. STATISTICA revealed three‐dimensional (3D) graph showing that the first three coordinates of the PCoA had Eigen values of 11.79, 6.89, and 6.12 with variances of 27.68%, 16.19%, and 14.33%. This 3D graph retrieved from STATISTICA (Figure 6a and Figure S3) showed the clustering of only five groups. The first group (Group 1) showed grouping of the Gambella‐Abol population, Group 2 contained the SNNPs‐Konta population, Group 3 had populations from the Kemash Zone of the BGR and from the West Wollega and Dabu Hena Zones of Oromia Region, Group 4 contained Assosa Zone populations, and Group 5 contained Metekel Zone populations. Populations from Group 3 could not be differentiated from one another. Populations of Assosa, Bambasi, and Kurmuk from the Assosa Zone of the BGR (Group 4) also clustered together. XLSTAT, on the other hand, categorizes into four groups. Group I and Group II both in the same sign (+/+ and −/−) were among the samples with relatively least diversity apart from the Dabu Hena population. Groups III and IV of opposite signs (either ± or ∓) were the most diverse samples and the most distant from one another. Apart from samples from the Assosa Zone, all populations showed their own cluster based on their geographical location. The populations were grouped into 11 clusters and XLSTAT 3D graph PCoA analysis (Figure 6b) yielded more detailed information than the 3D graph retrieved from STATISTICA.

### 3.7 Admixture analysis

The Bayesian approach‐based assignment of the 130 individual clumps to different populations and determination of their population structure (Evanno et al., 2005), using STRUCTURE outputs, predicted *K* = 2 for 13 populations for whole samples and *K* = 11 for 12 populations (excluding Gambella samples) to be the most likely number of clusters (Figures S1 and S2). Based on this value, Clumpak result (bar plot) showed little admixtures indicating geographic origin based structuring of populations.

## 4 DISCUSSION

The ultimate goal of this study is to develop genomic tools and resources with the possibility of contributing to development strategies for effective conservation and sustainable use of the *O. abyssinica* of Ethiopia for ecological and economic gains. So far, however, no studies have reported on its molecular genetic diversity. Hence, as a first step toward this goal, we have sought a greater understanding of the genetic diversity profile at the species and population level through an examination and assessment of 13 *O. abyssinica* populations using 19 ISSR markers. These molecular markers have been successfully utilized for assessing the genetic diversity and revealed a remarkable molecular genetic diversity among the Ethiopian lowland bamboo populations found in the country's different geographic locations.

### 4.1 ISSR markers for the genetic polymorphism in *O. abyssinica* populations

Out of the total 348 scorable bands produced with a total of 19 primers, 294 bands were polymorphic. In terms of the number of polymorphic fragment detected and percentage of polymorphic loci per class of primer, 3′ anchored dinucleotides were found to be superior. While the tri‐nucleotide primers generated 32 bands, tetra‐nucleotide generated 39 bands, and the penta‐nucleotide primers generated 56 bands, of which 25, 29, and 45 were polymorphic loci, respectively. Based on the anchorage property of the primers, 5′ anchored primers showed the poorest polymorphism with 36, 21, and 58.33 NSB, NPL, and PPL, respectively. Generally, anchored primers with NSB (221), NPL (195), and PPL (88.24) showed better NSB, NPL, and PPL than unanchored primers with values of 127, 99, and 77.95 respectively. This investigation, like those of Ng and Tan (2015) and Tarinejad, Sofalian, Valizadeh, and Shams (2015), showed anchored primers in general and 3′ anchored and dinucleotides ISSR primers in particular revealed better polymorphism than unanchored and other repeat motifs of ISSR markers for the study of *O. abyssinica* populations collected across Ethiopia. In terms of bands specificity, the highest numbers of private bands observed in Gambella, moderate in Koyshe, and almost none in other populations makes the Gambella population isolated and different from other *O. abyssinica* populations in the country.

The choice of molecular markers largely depends on the level of polymorphism to be detected and their genomic coverage rather than on the technology used to generate the markers. Estimates of marker‐based selection depend on the linkage of the genomic region and the marker itself. Because highly informative markers can reduce the amount of genotyping required for inference of ancestry, it is desirable to measure the extent to which specific markers contribute to this inference (Amom et al., 2020; Rosenberg, Li, Ward, & Pritchard, 2003). Several approaches have been developed previously for measuring polymorphism information, but a user‐friendly platform to calculate this information is missing or otherwise inaccessible (Nagy et al., 2012). The iMEC (marker efficiency calculator) created by a group of researchers (Amiryousefi, Hyvönen, & Poczai, 2018) is coded in R and is available as a web application helps to detect markers for lots of genetics researches. For the present study, the highest PIC, MI, *H*, and *D* were shown by anchored primers in general and by 3′ anchored and dinucleotide repeats in particular. Amom et al., (2020) using 10 ISSR primers for the genetic relationship study of five native and economical important bamboos of Northeast India, found that PIC values ranged from 0.271 to 0.454 with an average PIC value of 0.350 per primer. Penta and tri‐nucleotides as well as 5′ anchored primers showed the lowest *H*, PIC, and *D* values. Like the studies of Tarinejad et al. (2015) and Ng and Tan (2015), anchored and dinucleotide repeat primers showed more polymorphism and discriminating power than unanchored primers. It is noteworthy that dinucleotide repeats, anchored either at the 3′ or 5′ end, usually revealed high polymorphism and the primers anchored at the 3′ end gave clearer banding patterns compared to those anchored at the 5′ end (Joshi, Gupta, Aggarwal, Ranjekar, & Brar, 2000; Pradeep Reddy, Sarla, & Siddiq, 2002; Tarinejad et al., 2015).

### 4.2 Genetic differentiation and population structure

The high number of alleles and high polymorphism are very important for the correct estimation of the genetic diversity of germplasm. The degree of polymorphism showed the extent of diversity and effectiveness of the markers (Chesnokov & Artemyeva, 2015), and consequently, polymorphic information is related to expected heterozygosity and is usually determined from allele frequency. In the present study, the largest NPL (164 and 158), the highest PPL (47.13% and 45.40), *H* (0.2193 ± 0.2383 and 0.2165 ± 0.2407), and *I* (0.3088 ± 0.3330 and 0.3035 ± 0.3360) were found in the Guba and Dabu Hena populations. The lowest values of NPL, PPL, *H*, and *I* were observed in the Yasso, Abol and Kurmuk populations.

The overall NPL (294), PPL (84.48%), *H* ± *SD* (0.2702 ± 0.1945), *I* ± *SD* (0.4061 ± 0.2595), Na (1.8448 ± 0.3626), and Ne (1.4744 ± 0.3960) were recorded in the current study of *O. abyssinica* populations based on 19 ISSR markers. Compared to other bamboo species, the *O. abyssinica* populations in Ethiopia showed higher genetic diversity with *H* = 0.2702 ± 0.1945 and *I* = 0.4061 ± 0.2595. The *H* = 0.0418 ± 0.0456 and *I* = 0.0624 ± 0.0651 were recorded in Chinese giant bamboo (*Dendrocalamus giganteus*) population with low genetic diversity and high genetic differentiation (Tian et al., 2012). The *H* (0.175 ± 0.152) and *I* (0.291 ± 0.209) were observed in the *Dendrocalamus hamiltonii* population of the northeast Himalayas with low genetic diversity and a moderate level of genetic differentiation (Meena, Bhandhari, Barhwal, & Ginwal, 2019). High genetic variation at the species level with *H* = 0.1939 and *I* = 0.3218 were recorded in *Melocanna baccifera* (Roxb.) Kurz: bamboo of Manipur, Northeast India (Nilkanta et al., 2017). A high level of genetic diversity with *H* = 0.219 and *I* = 0.349 was also reported in *Dendrocalamus membranaceus*, a declining bamboo species in Yunnan, China, as based on ISSR analysis (Yang et al., 2012). Therefore, this tells us there is high genetic diversity and differentiation in *O. abyssinica* populations collected throughout Ethiopia. The Metekel Zone of BGR samples could be considered to possess higher genetic variation while Gambella, and SNNPs populations possess lower genetic variation when compared to other, indicating that these populations were subjected to genetic isolation.

Additionally, the present study on *O. abyssinica* recorded the total genetic diversity (*H*_t_ = 0.2708 ± 0.0379), genetic diversity within populations (*H*_s_ = 0.2047 ± 0.0327), the relative magnitude of genetic differentiation among populations (*G*_st_ = 0.2442), and estimate of gene flow among populations (*N*_m_ = 1.5474). The *H*_t_ = 0.1961, *H*_s_ = 0.1639, *G*_st_ = 0.1942, and *N*_m_ = 2.5455 with high genetic diversity was also recorded in *Melocanna baccifera* (Roxb.) Kurz: using seven ISSR primers (Nilkanta et al., 2017). The *H*_t_ = 0.167, *H*_s_ = 0.138, *G*_st_ = 0.247, and *N*_m_ = 1.52 with a low level of genetic diversity were also recorded in *D. hamiltonii* using 17 SSR markers (Meena et al., 2019).

### 4.3 Levels of genetic diversity among and within populations

Most bamboo populations are established through vegetative propagation. Sometimes, a population might be of a single rhizome clonally extended to a vast area. Hence, all individuals of a population might have similar or nearly similar genetic makeup (Bamboo Phylogenetic Group (BPG), 2012). The genetic similarity might result in total loss of the population if a certain disease or pest arises. Flowering creates an opportunity for gene mix up in the next generation since seeds can have more genetic variability. Bamboo is an out‐crossing wind‐pollinated plant where there is high gene random mix up, although flowering is an extremely rare event, sometimes only occurring once in life. As bamboos are mostly multiplied clonally, a single plant can form huge populations over time (Miyazaki, Ohnishi, Takafumi, & Hiura, 2009). This may follow a deficit of genetic variation within a population (Wong, 2004).

Our results of AMOVA with respect to grouping and without grouping populations are consistent with several works on bamboo. A study by Li et al. (2020) on IRAP marker‐based genetic diversity and population structure of 58 *Phyllostachys* accessions (Asian bamboo) using 16 primers reported that 75% of variation was within the population and 25% among the populations. The findings of Meena et al. (2019) on 19 populations of *D. hamiltonii using* 17 SSR markers indicated an AMOVA result of 16.53% among populations and 83.47% within populations (assuming no hierarchical grouping), and 6.28% among groups, 12.45% among populations within the group, and 81.26% within populations in four hierarchical groups. Nilkanta et al. (2017), who used five ISSR markers on *M. baccifera* (Roxb.) Kurz: bamboo of Manipur, Northeast India, found an AMOVA of 22% among populations and 78% within populations. Yang *et al*.'s study (2012) on *D. membranaceus* using 10 ISSR markers also reported an AMOVA result of 78.95% within the population and 21.05% among populations. Again, Attigala, Gallaher, Nason, and Clark (2017) on the study of genetic diversity and population structure of the threatened temperate woody bamboo *Kuruna debilis* (Poaceae: Bambusoideae: Arundinarieae) from Sri Lanka using 12 microsatellite loci reported an AMOVA result of 8.35% among groups, 7.52% among populations within groups, and 84.13% within populations. But compared to the above work on different bamboo species, the within‐population percentage of 61.05% for *O. abyssinica* is smaller.

On the other hand, *F*_SC_ = 0.10486, *F*_ST_ = 0.38949 and *F*_CT_ = 0.31797 in seven groups and *F*_ST_ = 0.36354 without grouping were recorded from *O. abyssinica* populations of Ethiopia. *F*_SC_ = 0.132, *F*_ST_ = 0.187, and *F*_CT_ = 0.062 in four groups and *F*_ST_ = 0.165 without grouping were reported from *D. hamiltonii* (Meena et al., 2019). Higher population divergence or differentiation was observed in *D. hamiltonii* (*F*_ST_ = 0.165), and very great or significant genetic differentiation with *F*_ST_ = 0.36354 was recorded in *O. abyssinica* populations. For the interpretation of *F*_ST_, it has been suggested that a value lying below 0.05 indicates little genetic differentiation; a value between 0.05 and 0.15, moderate differentiation; a value between 0.15 and 0.25, great differentiation; and values above 0.25, very great or significant genetic differentiation (De Vicente, Lopez, & Fulton, 2004; Wright, 1984). However, for interpretation through comparisons with earlier findings, another widely used measure of genetic differentiation such as *G*_ST_ was also considered along with *F*_ST_. The measure of genetic differentiation (*G*_ST_ = 0.2442) recorded in *O. abyssinica* was comparable to those of another bamboo species such as *D. hamiltonii* (*G*_ST_ = 0.247) (Meena et al., 2019), *D. membranaceus* (*G*_ST_ = 0.252) (Yang et al., 2012), and *M. baccifera* (*G*_ST_ = 0.194) (Nilkanta et al., 2017).

Many factors can determine the genetic structure and differentiation of plant populations, including reproductive biology, natural selection, genetic isolation or genetic drift, geographic distribution range, and gene flow (Hogbin & Peakall, 1999; Loveless & Hamrick, 1984; Schaal, Hayworth, Olsen, Rauscher, & Smith, 1998). Many ISSR, RAPD, and sequence‐tagged microsatellite sites (STMS)‐based genetic analyses showed that long‐lived, out‐crossing taxa retained most of their genetic variability within populations (Meena et al., 2019; Nilkanta et al., 2017; Nybom, 2004). The woody bamboos have a long vegetative phase of 20–150 years and typically a species with one of the greater longevities of the grass family (Ma et al., 2013). As one of those critical influences, the out‐crossing of a plant species tends to explain 10%–20% of the genetic variation among populations, whereas the selfing of a species leads, on average, to 50% variation between populations (Miyazaki et al., 2009). *Oxytenanthera abyssinica* can reproduce via seed in the wild, although this phenomenon is rare, and the rate of seed setting is low (Embaye, 2000; Ensermu et al., 2000; Zhao et al., 2018). Furthermore, studies on floral biology have indicated that *O. abyssinica* is likely anemophilous and prone to being an out‐crosser (Thakur, Barthwal, & Ginwal, 2016), which also was supported by the genetic differentiation (*G*_ST_ = 0.2442) that was similar to the average of out‐crossing species (*G*_ST_ = 0.22) (Nybom, 2004).

### 4.4 Clustering and admixture analysis

ISSR markers analysis for the genetic relationship study of five native and economical important bamboos (Amom et al., 2020), for the genetic diversity and structure of *Dendrocalamus hamiltonii* (Meena et al., 2019) and for the population genetic study of *Melocanna baccifera* (Roxb.) Kurz (Nilkanta et al., 2017), revealed that most of the populations were clustered in accordance with their geographical distribution and location. In our results, populations were also clustered in accordance with their geographical distribution and location. Most primer combinations in terms of nucleotide repeats and anchorage property showed almost similar clusters the overall assessment. But tri‐ and penta‐nucleotide repeat ISSR primer and anchored primers resulted in the poorest tree topology. The reasons that tri and penta‐nucleotides reveal the poorest information may be due to the availability of SSRs in small numbers when compared to other repeats motifs (Zhao et al., 2015).

Admixture results via STRUCTURE and web‐based data retrieval from structure harvester and CLUMPAK with and without Gambella samples showed different delta *K* values (*K* = 2 and *K* = 11) with an optimum population structure consisting of two and 11 sub‐populations. The delta *K* = 11 value is in agreement with the result of XLSTAT clustering the Ethiopian lowland bamboo into their geographic location. This implies that samples collected from the Gambella Region are different from others and may indicate a possible existence of additional bamboo species in the country.

## 5 CONCLUSION

The aim of the present study was to explore the extent of genetic diversity, population structure, and gene flow analysis of *O. abyssinica* populations and to devise mechanisms for effective identification, conservation and sustainable use of bamboo resources in Ethiopia using ISSR markers. Although a relatively high level of genetic diversity exists at species level, and a relatively low genetic differentiation was observed at the population level, most of the diversity was distributed within the populations, and cluster analysis grouped the populations into sharply distinct clusters, all of which could be attributed to the plant's cross‐pollination nature and long‐standing presence in the area. Using these ISSR markers, we found strong evidence linking geographic origin with diversity. The Metekel Zone in particular is in need of a conservation strategy, as it was found to have the most diverse population and samples from the Gambella Region were found to be different from those taken from others regions, indicating the availability of additional bamboo species in the country. Though the present investigation yielded some information on the genetic diversity of *O. abyssinica* populations in Ethiopia using ISSR markers, there is a need for further critical work involving molecular markers giving greater genome coverage to improve our understanding of genetic diversity in bamboo at the species and population level.

## CONFLICT OF INTEREST

The authors declare no conflict of interest.

## AUTHOR CONTRIBUTIONS

**Oumer Abdie Oumer:** Conceptualization (lead); data curation (lead); formal analysis (lead); funding acquisition (lead); investigation (lead); methodology (lead); project administration (lead); resources (lead); software (lead); supervision (lead); validation (equal); visualization (lead); writing–original draft (lead); writing–review and editing (lead). **Kifle Dagne:** Conceptualization (equal); data curation (equal); formal analysis (equal); investigation (equal); methodology (equal); project administration (equal); resources (equal); software (equal); supervision (equal); validation (equal); visualization (equal); writing–original draft (equal); writing–review and editing (equal). **Tileye Feyissa:** Conceptualization (lead); data curation (equal); formal analysis (lead); investigation (equal); methodology (equal); software (equal); supervision (equal); validation (equal); visualization (equal); writing–original draft (equal); writing–review and editing (equal). **Kassahun Tesfaye:** Conceptualization (lead); data curation (equal); formal analysis (lead); funding acquisition (equal); investigation (equal); methodology (lead); project administration (lead); resources (lead); software (equal); supervision (equal); validation (equal); visualization (equal); writing–original draft (equal); writing–review and editing (lead). **Jayaraman Durai:** Funding acquisition (lead); project administration (supporting); writing–original draft (supporting); writing–review and editing (equal). **Muhammad Zeeshan Hyder:** Conceptualization (equal); data curation (equal); funding acquisition (equal); investigation (equal); methodology (equal); project administration (equal); resources (equal); software (equal); supervision (equal); validation (equal); visualization (equal); writing–original draft (equal); writing–review and editing (equal).

### Open Research Badges

This article has been awarded <Open Data, Open Materials, Preregistered Research Designs> Badges. All materials and data are publicly accessible via the Open Science Framework at https://doi.org/10.5061/dryad.b5mkkwh8z.

## Supporting information

Fig S1‐S3

## Data Availability

*Oxytenanthera abyssinica* (A. Rich.) Munro; lowland bamboo (Poaceae, Bambusinea) in Ethiopia: Genetic diversity, population structure and gene flow analysis submitted with https://doi.org/10.5061/dryad.b5mkkwh8z.
