# Supplementary material for: Genetic diversity, population structure, and gene flow analysis of lowland bamboo [Oxytenanthera abyssinica (A. Rich.) Munro] in Ethiopia
Source: Ecol Evol. 2020 Sep 20;10(20):11217–36. doi: 10.1002/ece3.6762 (PMC7593185; doi:10.1002/ece3.6762)
Supplement: Supplementary file 1 — Fig S1‐S3 [file ECE3-10-11217-s001.docx]

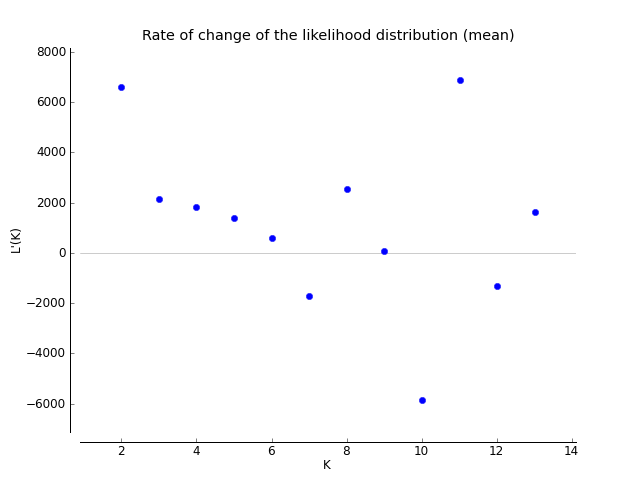

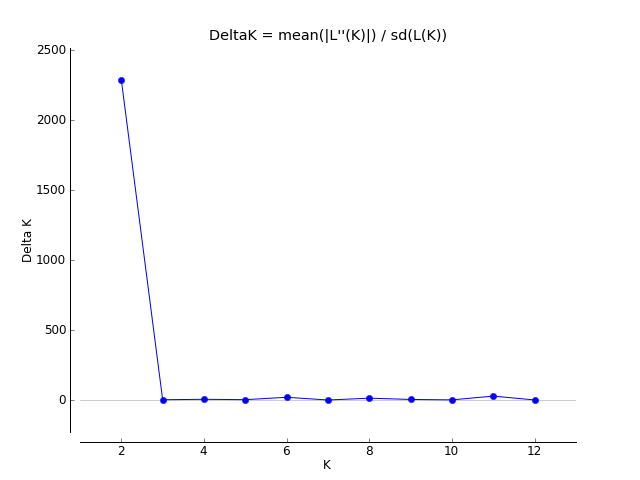

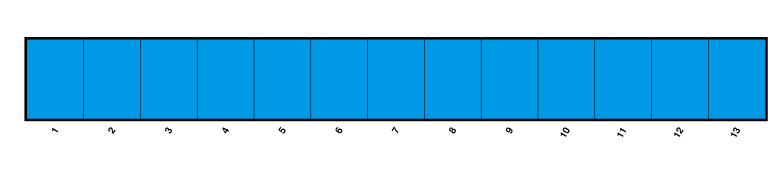


**B**

**A**

K = 13

K = 12

K = 11

K = 10

K = 9

K = 8

K = 7

K = 6

K = 5

K = 4

K = 3

K = 2

K = 1


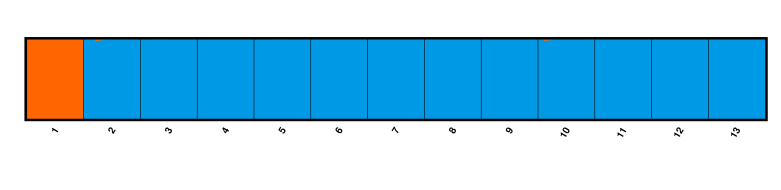


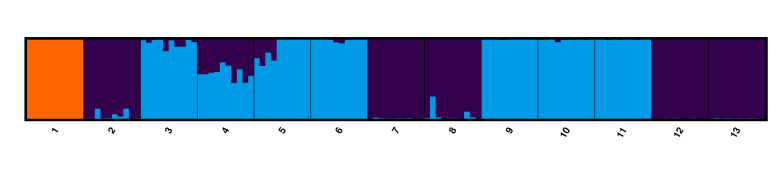


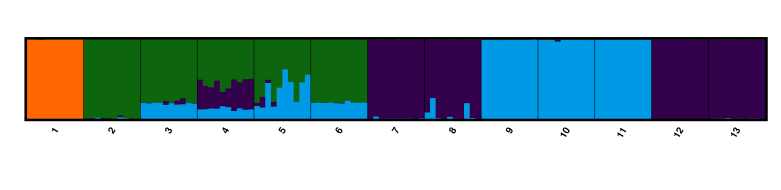


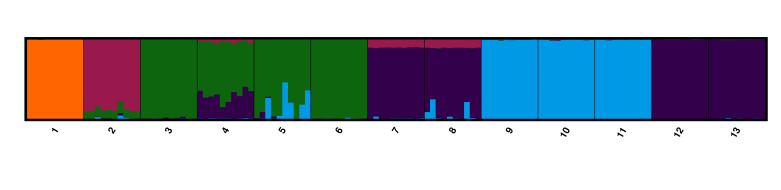


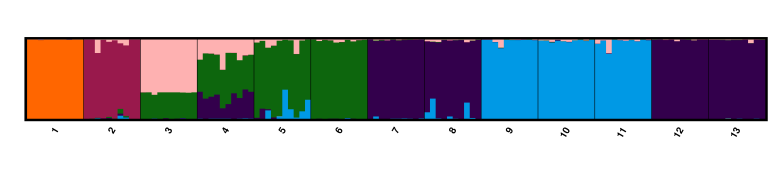


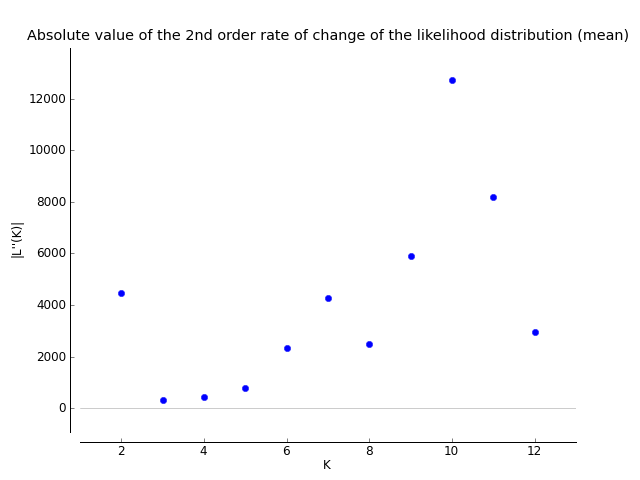

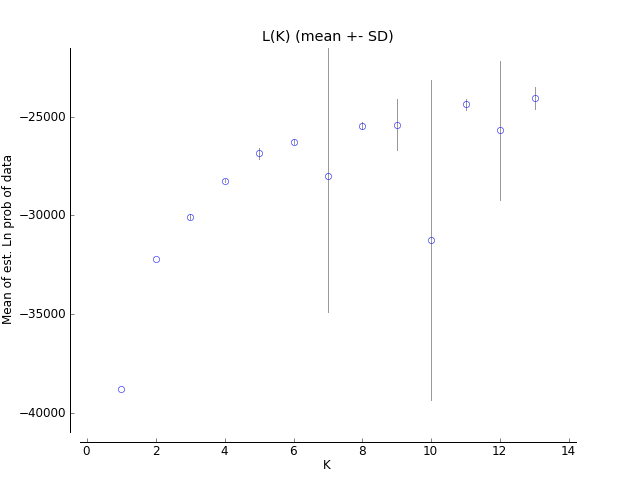

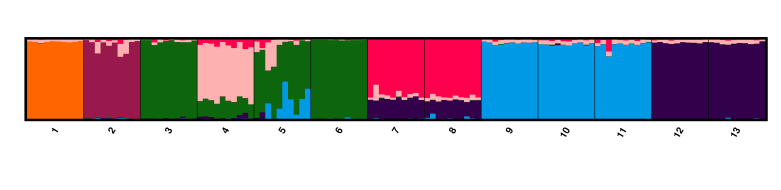


**D**

**C**


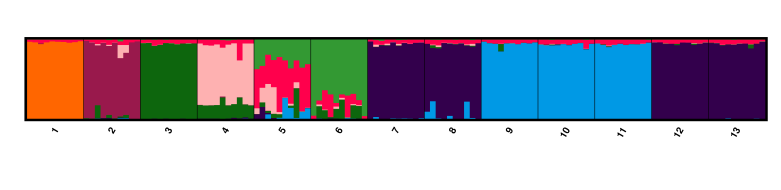


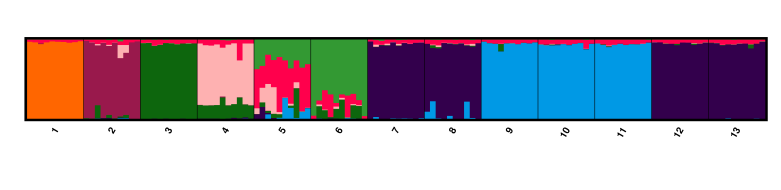


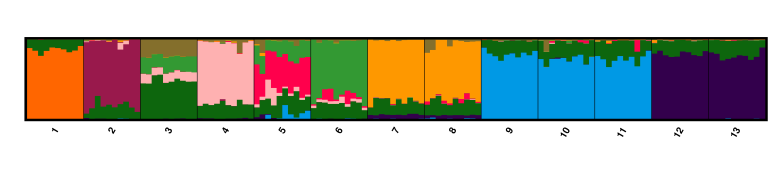


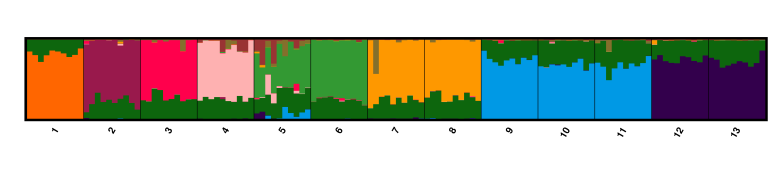


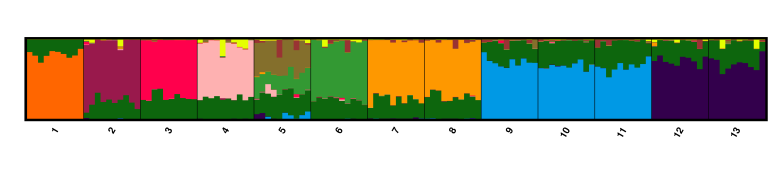


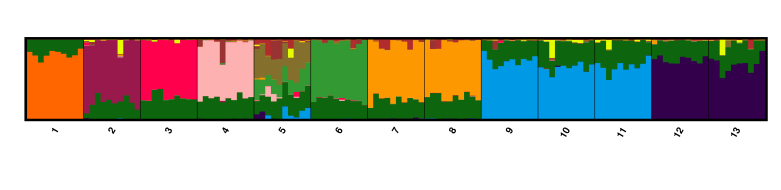


GGAM-Abol

SNNPs-Koys

BGM-Mand BGM-Dang

BGM-Guba

BGM-Pawe

BGK-Kema

BGK-Yass

BGA-Asso

BGA-Bamb

BGA-Kurm

ORWW-Gimb

ORBB-DabuH

**Figure 1:** STRUCTURE harvester and CLUMPAK Delta K value estimated using Evano *et al.* (2005) method and Bayesian model-based estimation of population structure for 130 Ethiopian lowland bamboos (*O. abyssinica*) based on ISSR markers in thirteen pre-determined populations. (**A**), ΔK = mean(|L”(K)|)/sd(L(K)). ΔK = 2 indicates the maximum K value; (B), rate of change of the likelihood distribution (mean); (C), mean of estimated Ln probability; (D), absolute value of the 2^nd^ order rate of change of the likelihood distribution (mean).


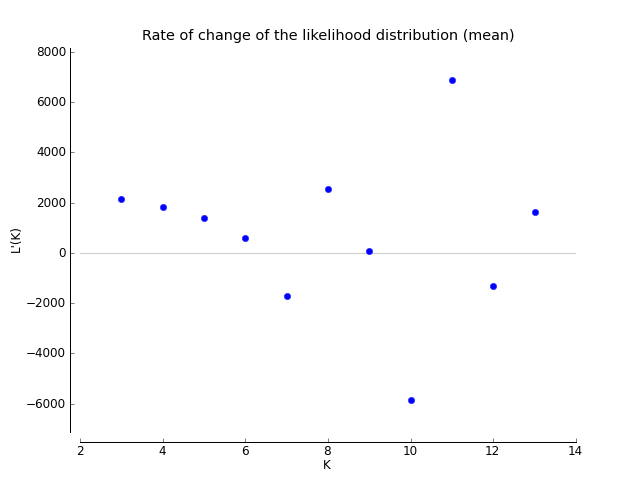

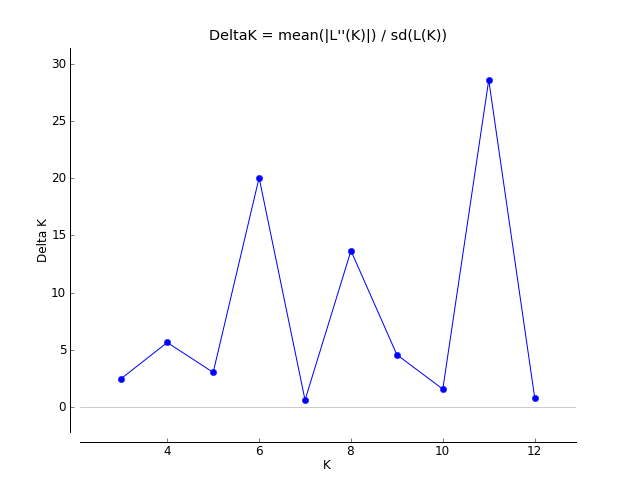

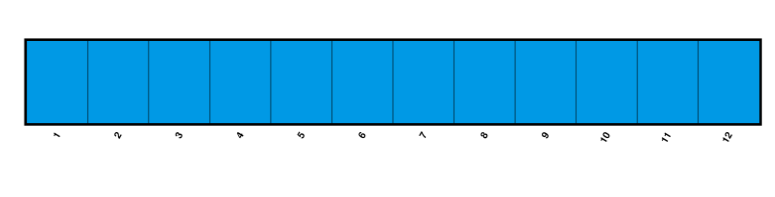


**B**

**A**

K = 12

K = 11

K = 10

K = 9

K = 8

K = 7

K = 6

K = 5

K = 4

K = 3

K = 2

K = 1


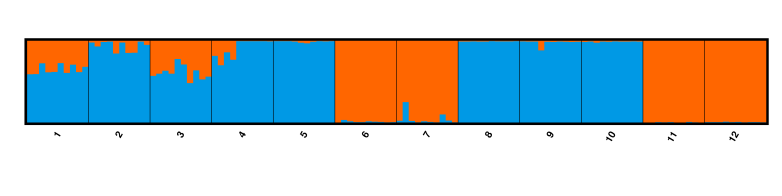


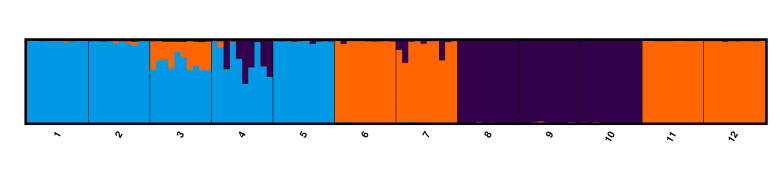


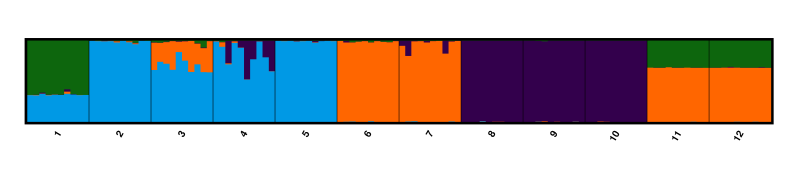


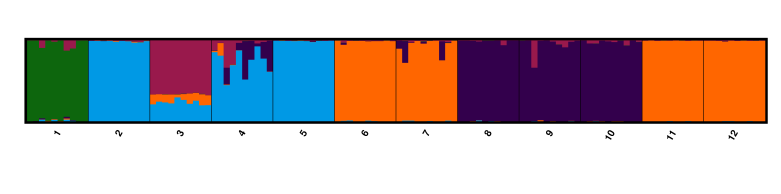


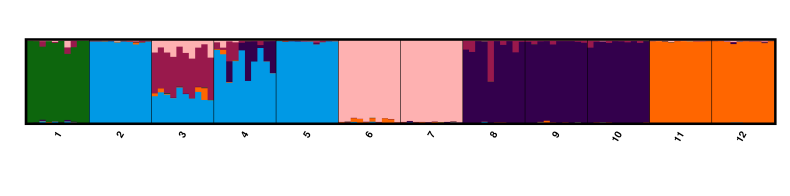


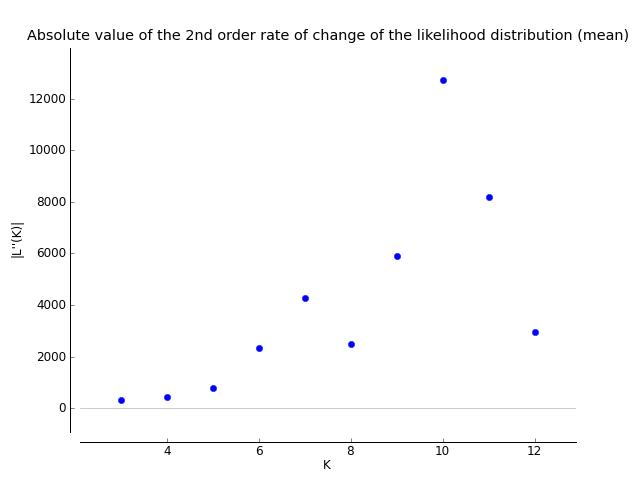

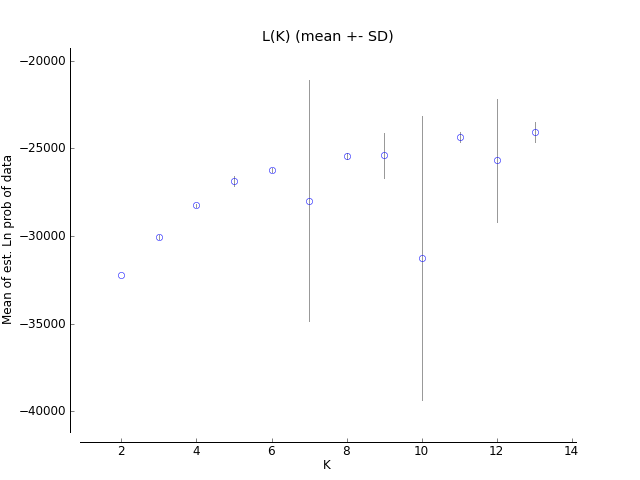

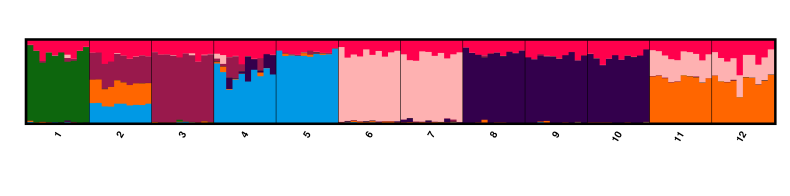


**C**

**D**


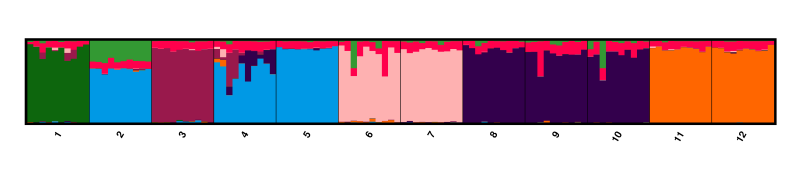


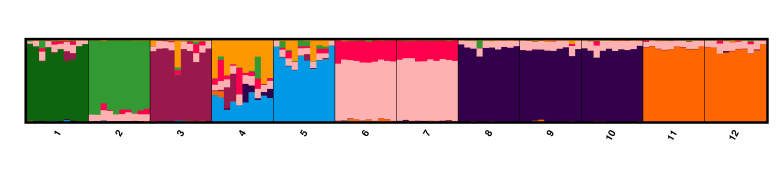


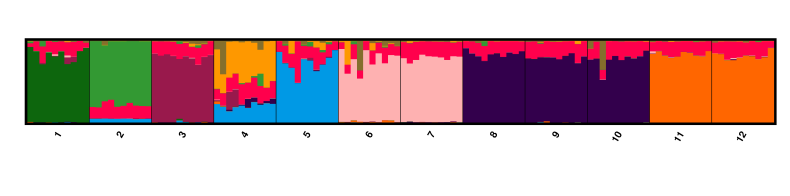


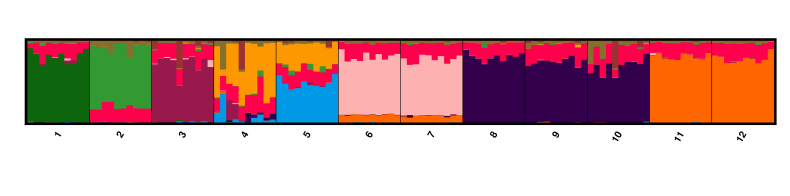


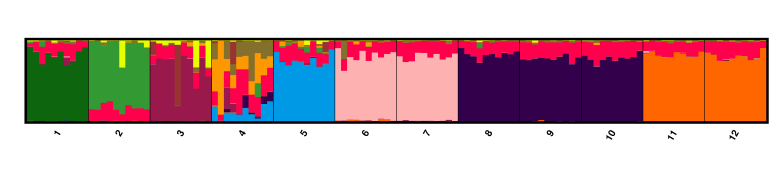


GAM-Abol

SNNPs-Koys

BGM-Mand BGM-Dang

BGM-Guba

BGM-Pawe

BGK-Kema

BGK-Yass

BGA-Asso

BGA-Bamb

BGA-Kurm

ORWW-Gimb

ORBB-DabuH

**Figure 2:** STRUCTURE harvester and CLUMPAK Delta K value estimated using Evano *et al.* (2005) method and Bayesian model-based estimation of population structure for 120 Ethiopian lowland bamboos (*O. abyssinica*) based on ISSR markers in twelve pre-determined populations (excluding Gambella samples). (**A**), ΔK = mean(|L”(K)|)/sd(L(K)). ΔK = 11 indicates the maximum K value; (B), rate of change of the likelihood distribution (mean); (C), mean of estimated Ln probability; (D), absolute value of the 2^nd^ order rate of change of the likelihood distribution (mean).

**Group 5**

**Group 4**

**Group 2**

**Group 3**

**Group 1**

**Figure** **3:** Three dimensional representation of principal coordinate analysis of genetic relationships among 130 individuals of 13 populations of Ethiopian lowland bamboo (*O. abyssinica*) inferred from similarity matrix using the Jaccard’s index at STATISTICA.
